# A hybrid deep learning framework for real‐time speckle reduction and image enhancement on portable ultrasound systems

**DOI:** 10.1002/mp.70620

**Published:** 2026-08-03

**Authors:** Hyunwoo Cho, Jaeseok Lee, Jongsoo Lee, Yangmo Yoo, Jinbum Kang

**Affiliations:** ^1^ Department of Electronic Engineering Sogang University Seoul South Korea; ^2^ Department of Urology, Urological Science Institute, College of Medicine Yonsei University Seoul South Korea; ^3^ Department of Biomedical Engineering Sogang University Seoul South Korea; ^4^ Department of Biomedical Software Engineering The Catholic University of Korea Bucheon South Korea

**Keywords:** deep neural network, hardware acceleration, portable ultrasound device, self‐supervised learning, speckle reduction, ultrasound imaging, unsupervised learning

## Abstract

**Background:**

Speckle patterns in ultrasound images often obscure anatomical details, leading to diagnostic uncertainty. Recently, various deep learning‐based techniques have been introduced to effectively suppress speckle; however, their high computational costs pose challenges for low‐resource devices, such as portable ultrasound systems.

**Purpose:**

To address this issue, we introduce Edge Speckle Reduction and Image Enhancement (EdgeSRIE), a lightweight hybrid deep learning framework for real‐time speckle reduction and image enhancement in portable ultrasound imaging.

**Methods:**

The proposed framework consists of an unsupervised despeckling branch and a self‐supervised deblurring branch trained separately in sequence with AdamW (learning rate 1 × 10^−^^4^) and an *L2* loss. The integrated model was converted to an 8‐bit deployment model using post‐training quantization on a low‐resource system‐on‐chip. Training used 1779 B‐mode images (BUSI and HC18), validation used 77 raw acquisitions (PICMUS and CUBDL), and external evaluation used 94 EdgeFlow UH‐10 bladder images. EdgeSRIE was compared with OSRAD, OBNLM, DIAE, DUNet, BRUNet, and USNet using contrast‐to‐noise ratio (CNR), speckle signal‐to‐noise ratio (SSNR), average gradient magnitude (AGM), and structural similarity index measure (SSIM); paired inference used two‐sided Wilcoxon signed‐rank tests with Holm correction and rank‐biserial and Hedges’ *g* effect sizes.

**Results:**

Across the four representative cases, post‐training‐quantized EdgeSRIE achieved the largest mean improvement in CNR (64.9 ± 21.0%) and the smallest mean decrease in AGM (−16.6 ± 33.2%). SSNR (109.8 ± 45.8%) was in the OBNLM‐led range, whereas SSIM remained slightly lower than OSRAD and OBNLM on its native [0, 1] scale. The deployed model contained 17.67K parameters and reached 64.10 frames/s on the target hardware. In the case‐matched analysis (*n* = 8), EdgeSRIE showed Holm‐corrected significant advantages over all six baselines in CNR, over five of six in SSNR, and over four of six in AGM. For SSIM, EdgeSRIE was significantly higher than the four deep learning baselines but lower than OSRAD and OBNLM. Across the statistically significant comparisons, the magnitude‐based effect sizes were large (Hedges’ *g* ≥ 0.8).

**Conclusions:**

These results support the feasibility of EdgeSRIE as a compact, deployment‐oriented framework that balances speckle suppression, structural preservation, and real‐time execution for portable ultrasound imaging.

## 1 INTRODUCTION

Ultrasound (US) imaging is widely used in clinical practice because of its noninvasive nature, real‐time capability, and cost‐effectiveness.^1^ However, speckle patterns—arising from the constructive and destructive interference of backscattered waves from numerous scatterers within a resolution cell—often limit its diagnostic utility.^2^ Their granular appearance depends on the tissue structure and imaging parameters, including the transducer frequency and geometry.^3^ Although speckles can benefit certain applications, such as tissue characterization^4^, ^5^ and speckle motion tracking,^6^, ^7^ they generally degrade image contrast and may obscure lesion margins, cyst boundaries, lumen‐wall interfaces, thin intima‐media layers, and subtle tissue texture patterns, thereby complicating segmentation, registration, and computer‐aided detection.^8^, ^9^ Consequently, effectively suppressing speckle noise while preserving essential diagnostic details is crucial for improving image quality in US imaging.

Since speckle is caused by multiplicative noise rather than additive noise,^10^ simply using averaging filters or low‐noise electronics is insufficient for effective speckle suppression.^11^ To address this challenge, traditional speckle reduction techniques—which are based on local statistics, anisotropic diffusion, and nonlocal means—have been introduced.^12^, ^13^, ^14^, ^15^, ^16^, ^17^, ^18^, ^19^, ^20^ Despeckle filters employing local statistics (e.g., Lee,^12^ Frost et al.^13^ and Kuan et al.^14^) typically perform a weighted average in subregions to compute statistical measures over differing pixel windows. While these methods substantially reduce speckle noise, they can also suppress valuable diagnostic features. To mitigate these limitations, several anisotropic diffusion filters that solve a partial differential equation have been proposed to preserve tissue boundaries and important details while effectively suppressing speckle noise.^15^, ^16^, ^17^, ^18^ Notably, an oriented speckle reducing anisotropic diffusion (OSRAD) technique was introduced, wherein a diffusion matrix facilitates directional filtering along structures, thereby improving edge preservation relative to local statistical filters.^16^ In contrast to methods based on local statistics or diffusion models, the nonlocal mean filtering (NLM) algorithm,^19^ which evaluates the similarity among image patches to assign different weights, was refined for US imaging as the optimized Bayesian nonlocal means (OBNLM).^20^ This refinement demonstrated notable improvements in speckle noise suppression and edge preservation. However, the assumption of homogeneous local regions can be inappropriate for medical ultrasound imagery, and the blockwise nature of NLM leads to high computational complexity.

Although rule‐based speckle reduction can improve US image quality, it often requires extensive parameter tuning, resulting in subjective variations.^21^ To address these drawbacks without resorting to overly complex models, deep neural network (DNN)‐based approaches have been explored,^21^ including a despeckling residual neural network (DRNN) that outperformed conventional filters.^21^ The DRNN uses a ResNet‐based generator^22^ within a generative adversarial network framework.^23^ A modified version of PCANet combined with NLM filtering was used to extract robust features for enhanced despeckling.^24^ A joint beamforming and speckle reduction network was used to reconstruct B‐mode images from channel data,^25^ but varying transducer properties limit its clinical practicality. Another study evaluated five DNN architectures,^26^ among which a dilated‐convolution autoencoder (DIAE), a U‐Net–based denoising network (DUNet), and a batch‐renormalization U‐Net (BRUNet) outperformed traditional filters. A lightweight US despeckling network, US‐Net (USNet), was used to reduce speckle and enhance texture while maintaining low computational overhead.^27^ Since acquiring ground‐truth data is challenging, unsupervised frameworks have also emerged as popular options.^28^, ^29^ For example, S2S^28^ performs well without high‐quality references but relies on steered ultrafast imaging in the IQ domain, restricting its applicability to publicly available databases.^28^

Despite the promising potential of deep learning (DL) approaches in medical US imaging, their deployment in conjunction with portable US devices remains highly challenging due to computational constraints. Most conventional DNN architectures involve millions of parameters, resulting in substantial computational overhead.^30^ This overhead limit their feasibility on the low‐resource system‐on‐chip (SoC) platforms commonly used with portable medical imaging devices. Designed for critical scenarios such as emergency medicine and point‐of‐care diagnostics, these compact US systems have limited computational power and battery capacity, often leading to lower image quality than high‐end devices provide.^31^, ^32^, ^33^ Thus, robust speckle reduction and image enhancement techniques optimized for resource‐constrained environments are urgently needed. In recent years, several lightweight DNNs have been proposed to achieve real‐time speckle suppression and image enhancement. USNet,^27^ for example, effectively reduces speckle while maintaining relatively low computational complexity, making it suitable for conventional hardware. However, many of these lightweight DNNs still depend on performance‐intensive CPUs or GPUs,^27^, ^28^ rendering them unsuitable for truly portable, battery‐powered medical devices. Although some studies have deployed DL algorithms on low‐resource SoC platforms for various US imaging tasks,^34^, ^35^ no speckle reduction or image enhancement methods have been specifically designed and optimized for portable US systems. Therefore, developing advanced yet compact DNN frameworks is critical to bridging the gap between high‐quality speckle reduction and the practical limitations of portable US imaging.

To address this limitation, this paper introduces Edge Speckle Reduction and Image Enhancement (EdgeSRIE), a lightweight, hybrid DL framework for real‐time speckle reduction and image enhancement for portable US systems. With its dual branch design, encompassing an unsupervised despeckling branch and a self‐supervised deblurring branch, EdgeSRIE suppresses speckle patterns while preserving critical image details. To meet resource constraints, the dual‐branch network was designed to use fewer than 20K parameters, quantized to 8‐bit precision, and implemented on a low‐resource SoC via a hardware accelerator. The proposed framework was evaluated using minimally processed PICMUS in vivo raw‐acquisition data,^38^ CUBDL phantom data,^39^ and portable‐device bladder images. The present study focuses on engineering and image‐quality feasibility rather than formal reader‐based clinical validation.

## 2 METHODS

### 2.1 Overall framework

**FIGURE 1:**
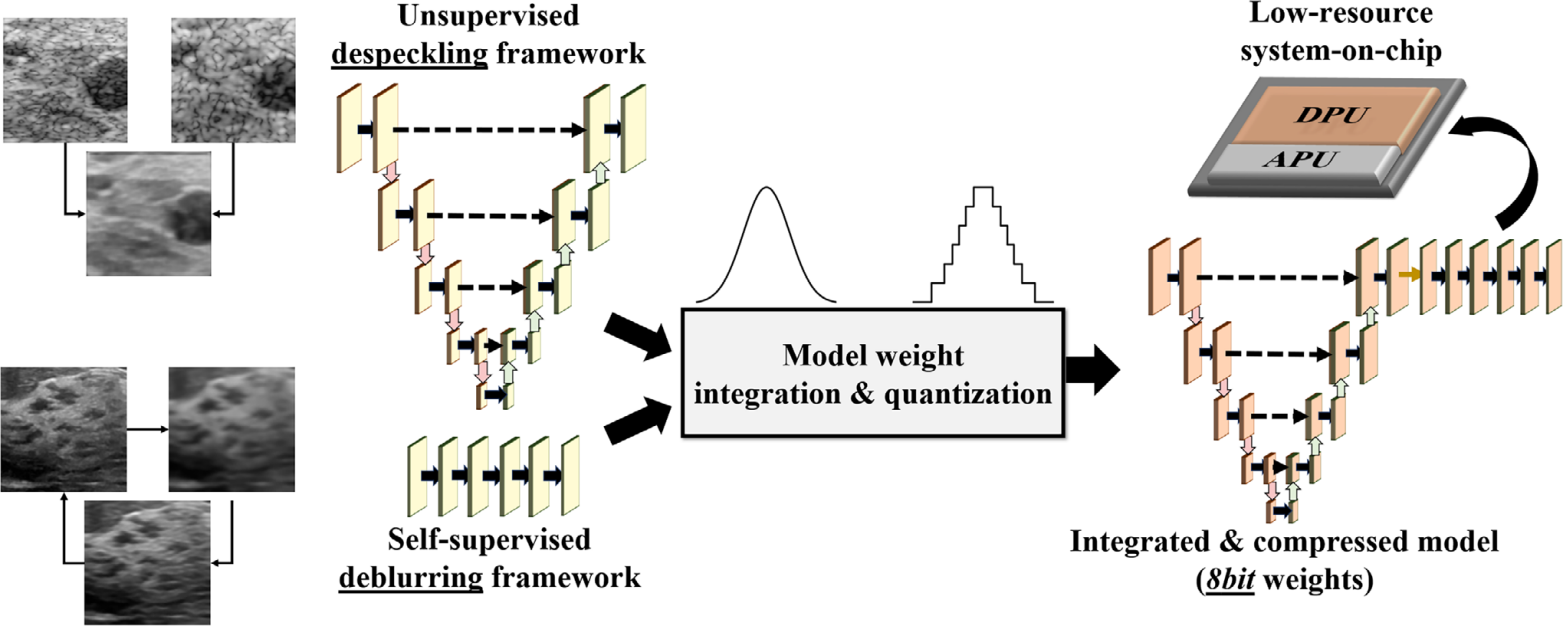
Overview of the proposed EdgeSRIE framework. The hybrid deep‐learning model consists of two complementary branches trained separately in sequence: An unsupervised branch for speckle reduction and a self‐supervised branch for image deblurring. After branch‐wise training, the two branches are integrated into a hybrid floating‐point model and then quantized from 32‐bit floating‐point to 8‐bit integer precision using post‐training quantization for deployment on a low‐resource SoC platform.

Figure 1 illustrates the overall workflow of the proposed EdgeSRIE framework, which is specifically designed for real‐time US speckle reduction and image enhancement on portable US devices. The EdgeSRIE framework integrates two complementary DL branches, an unsupervised despeckling branch and a self‐supervised deblurring branch, each targeting distinct aspects of US image quality enhancement. The two branches were trained separately in sequence and then integrated into a single hybrid model, rather than being jointly optimized from scratch. The integrated floating‐point model was subsequently converted to 8‐bit integer precision using post‐training quantization to reduce computational complexity for deployment on low‐resource SoC platforms.

### 2.2 Unsupervised framework for despeckling

**FIGURE 2:**
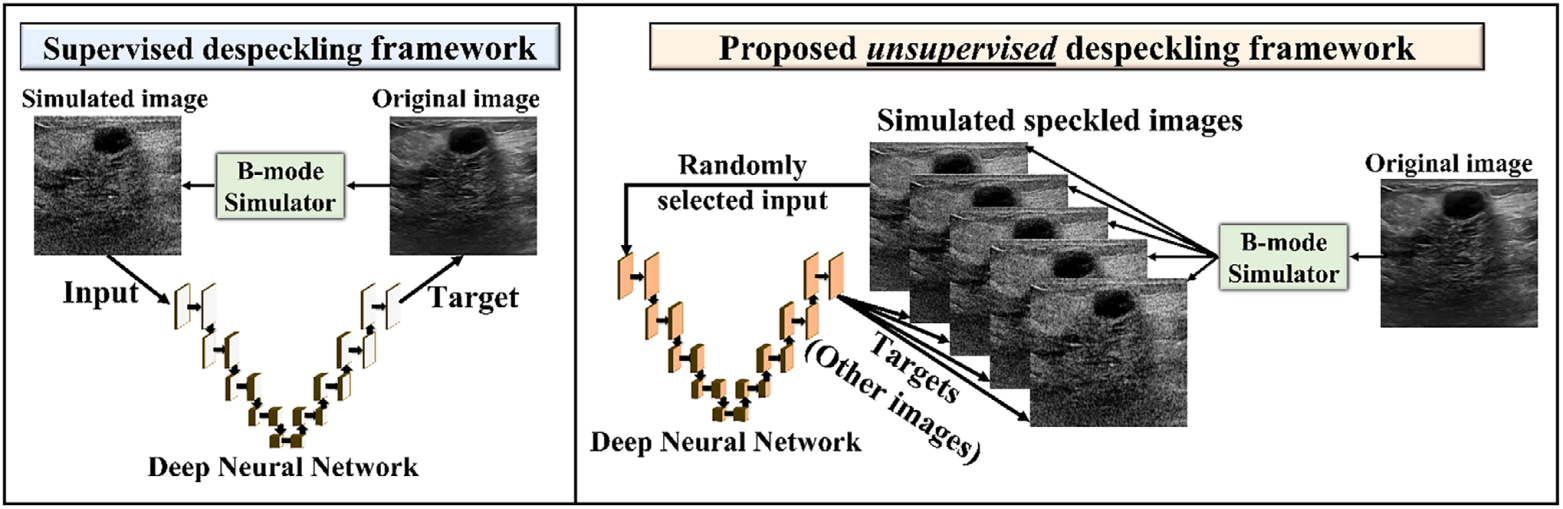
Comparison between the conventional supervised framework and the proposed unsupervised ultrasound despeckling framework. In conventional supervised approaches (left), a B‐mode simulator generates a single simulated speckle image from an original clean image, requiring matched pairs of input and reference data for network training. In contrast, the proposed unsupervised framework (right) leverages multiple simulated speckle images generated from a single original image. One randomly selected speckle image serves as the input, while the remaining speckle images act as indirect targets. This strategy eliminates the requirement for perfectly clean ground‐truth references, facilitating robust and generalized learning across diverse speckle patterns.

Figure 2 provides a comparison between the conventional supervised framework and our proposed unsupervised framework for US despeckling. Conventional supervised methods^26^, ^27^ typically generate artificially speckled images by applying random noise or simulation techniques, pairing each speckled image with a corresponding clean original image for training. In contrast, the proposed framework generates multiple speckle realizations from the same source image using a simplified B‐mode simulator. One realization is used as the network input, whereas the remaining realizations provide indirect supervision through structural consistency across differing speckle patterns.^36^ Because these realizations share the same underlying anatomy while differing stochastically, the shared structural component dominates the learning signal and realization‐specific fluctuations tend to average out across mini‐batches.

In this study, the simulator was used as a simplified forward B‐mode model for generating realistic speckle diversity rather than as a full‐wave acoustic solver. Following the standard convolution‐based formulation of B‐mode image formation, the normalized input image *I* was used as an image‐derived echogenicity‐related map. A stochastic field R was scaled by the speckle‐variance parameter $\sigma_{s}$, and a scattering‐like map was formed as

(1) $$S\;\left(x,z\right)=I\left(x,z\right)(1+\sigma_{s}\left(R\left(x,z\right)\right)$$

The effective point spread function was modeled as a separable lateral‐axial kernel

(2) $$h\;\left(x,z\right)=h_{x}\;\left(x;\sigma_{x}\right)h_{z}\left(z;\sigma_{z}\right)$$

where

(3) $$\begin{array}{cc} h_{z}\;\left(z;\sigma_{z}\right) & =\sin\left(k_{0}z\right)\exp\left(-\frac{z^{2}}{\left(2\sigma_{z}^{2}\right)}\right)\;,\\ h_{x}\;\left(x;\sigma_{x}\right) & =\exp\left(-\frac{x^{2}}{2\sigma_{x}^{2}}\right) \end{array}$$

The acoustic wavenumber was defined as

(4) $$k_{0}=\frac{2\pi f_{0}}{c}\;$$

**FIGURE 3:**
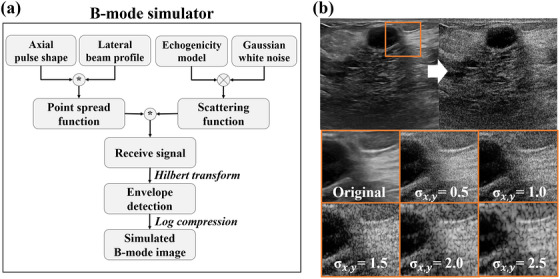
(a) Simplified forward B‐mode simulator used for generating realistic speckled ultrasound images. A stochastic scattering‐like map is formed from an image‐derived echogenicity‐related map and a random field, and an effective point spread function (PSF) is defined by axial and lateral kernels. The simulated receive signal is generated by separable convolution, followed by Hilbert‐transform‐based envelope detection and log compression. (b) Example of an original ultrasound image and a corresponding simulated speckled image. The magnified views illustrate that varying PSF‐related parameters yields multiple realistic speckle realizations from the same underlying structure.

The simulated receive signal was generated by convolving *S* with this kernel, followed by Hilbert‐transform‐based envelope detection, log compression, and brightness normalization to obtain the final speckled B‐mode‐like image (Figure 3).^37^

The simulator parameters were chosen to be representative of clinical B‐mode imaging on portable US systems following the standard convolution‐based B‐mode formation model.^37^ The center frequency was set to 10 MHz, sound speed to 1540 m/s, consistent with the conventional soft‐tissue assumption used in diagnostic US imaging.^37^ The axial and lateral Gaussian widths were sampled from {0.5, 1.0, 1.5, 2.0, 2.5} to emulate point‐spread‐function variation across depth and beam conditions,^37^ and the speckle‐variance parameter was fixed to 0.2 to reproduce fully developed, multiplicative speckle statistics.^2^, ^8^, ^9^ The log‐compression dynamic range was set to 30 dB, consistent with typical clinical B‐mode display settings.^37^ During unsupervised training, one simulated speckled image was randomly selected as input and the remaining realizations from the same source served as indirect targets. Generalization across acquisition and device conditions was evaluated empirically on the independent PICMUS^38^ and CUBDL^39^ benchmarks and the external UH‐10 portable‐device cases (Figure 3).

### 2.3 Self‐supervised framework for deblurring

**FIGURE 4:**
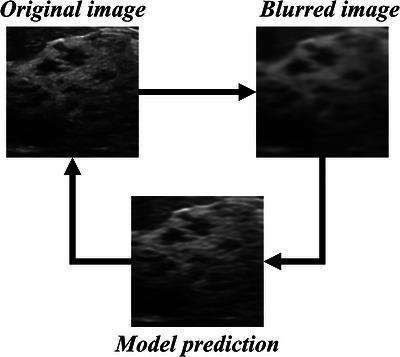
Illustration of the proposed self‐supervised deblurring framework. A reference image produced by the pretrained despeckling branch is synthetically degraded using blur and contrast compression, and the deblurring branch is trained to restore the corresponding reference image. This branch‐wise self‐supervised design enables image sharpening without requiring externally labeled paired sharp images.

Moreover, portable US images can suffer from blur due to reduced scanline density, limited transducer performance, or tissue motion during handheld operation in point‐of‐care settings. Despeckling algorithms can also introduce mild smoothing. To address these issues, the deblurring branch was trained in a self‐supervised manner: a degraded input was synthesized by applying random Gaussian blur and contrast compression to the corresponding pretrained despeckled reference, and the branch was trained to restore that reference using an *L2* reconstruction loss (Figure 4).

### 2.4 Network architecture and real‐time integration

**FIGURE 5:**
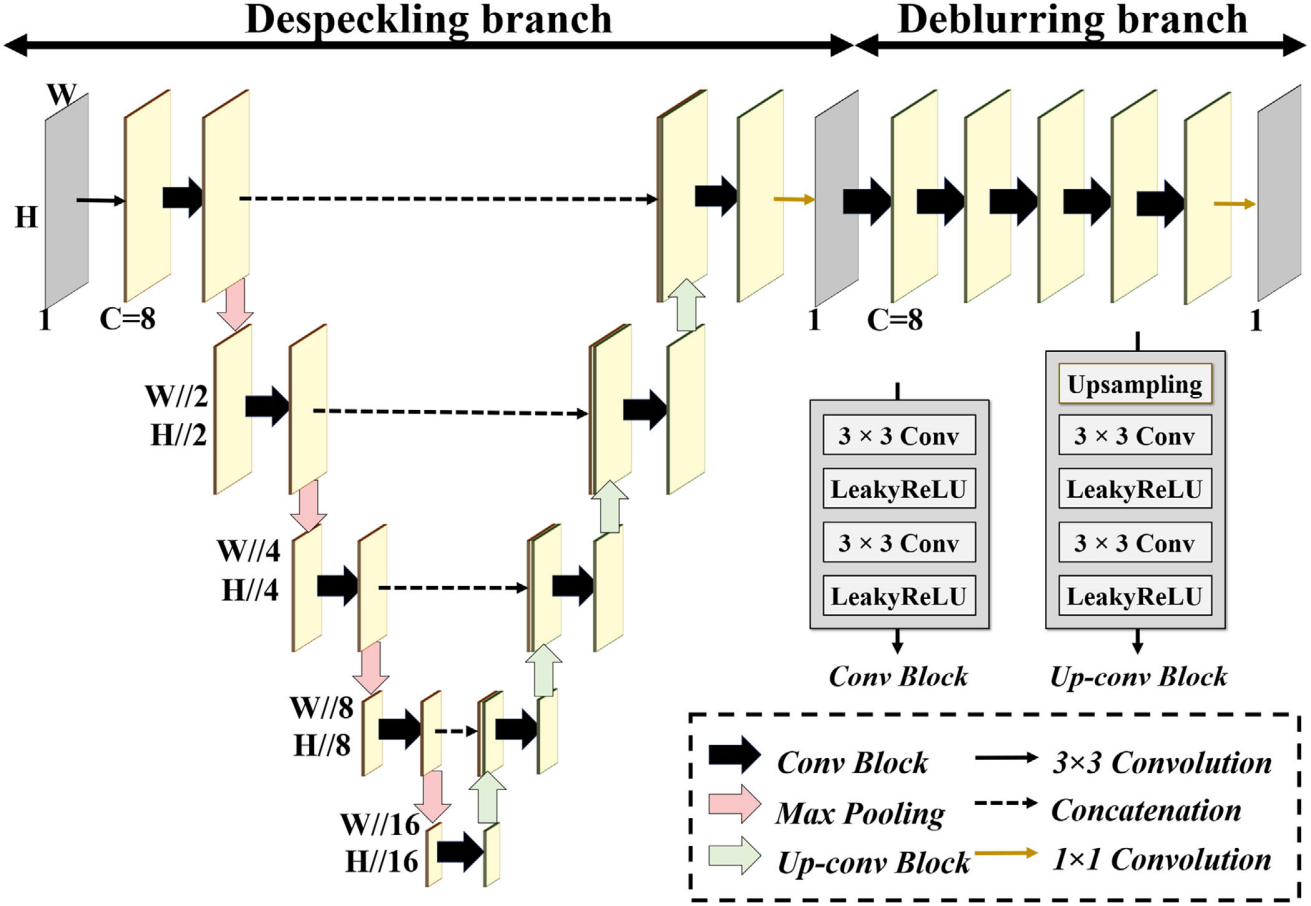
Proposed dual‐branch network architecture comprising a despeckling branch (left) and a deblurring branch (right). The despeckling branch is a lightweight U‐Net composed of five convolutional blocks, four max‐pooling stages, four upsampling stages, and skip connections. The deblurring branch consists of five sequential convolutional blocks without multiscale downsampling. Both branches produce single‐channel outputs, and the channel width is fixed to *C* = 8 for portable deployment.

Figure 5 illustrates the dual‐branch network architecture designed to address both speckle reduction and deblurring in US images. The despeckling branch is a lightweight U‐Net‐like architecture^40^ that extracts multiscale features through five convolutional blocks and four max‐pooling stages, followed by four upsampling staages with skip connections. In contrast, the deblurring branch is a lightweight local restoration network composed of five sequential convolutional blocks without multiscale downsampling or upsampling. In both branches, the channel width was fixed to *C* = 8 to minimize the total parameter count. The full model contains 17.67K parameters and 564.14 M floating‐point operations, making it suitable for deployment on resource‐constrained portable hardware.

**FIGURE 6:**
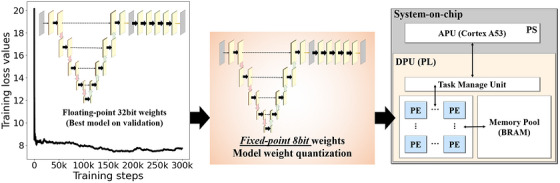
Training, integration, quantization, and deployment pipeline of the proposed network on a low‐resource SoC. The unsupervised despeckling and self‐supervised deblurring branches were trained separately in sequence, integrated into a hybrid floating‐point model, and then converted to an 8‐bit deployment model using post‐training quantization.

Figure 6 illustrates the training, integration, quantization, and deployment pipeline of the proposed EdgeSRIE framework on a low‐resource SoC platform. First, the unsupervised despeckling branch was trained in 32‐bit floating‐point precision using simulator‐generated multi‐speckle inputs. Second, the deblurring branch was trained separately using synthetically blurred versions of the corresponding pretrained despeckled references. After these two sequential stages, the learned branch weights were integrated into a single hybrid floating‐point model. The integrated model was then converted to 8‐bit precision using post‐training quantization^41^ and implemented with the Xilinx Vitis AI framework^42^ on an embedded SoC comprising an application processing unit (APU) and a deep‐learning processing unit (DPU).

### 2.5 Dataset preparation and experimental setup

To train and validate the proposed architecture, both publicly available B‐mode image datasets and raw‐acquisition US datasets were used. Rather than splitting a single dataset or subject across training and testing, the datasets were assigned to mutually exclusive roles at the source level. BUSI^43^ and HC18^44^ were used exclusively for model development (despeckling and deblurring training) because they provide diverse anatomical structures and image textures suitable for simulator‐based training; PICMUS^38^ and CUBDL^39^ served as held‐out raw acquisitions for representative evaluation, with checkpoint selection monitored on a representative held‐out acquisition; and the portable EdgeFlow UH‐10 bladder images served as an external test set acquired on a different device and cohort. Consequently, no subject, acquisition, or probe sweep was shared across roles, so cross‐sample leakage cannot occur; the per‐image and per‐acquisition labels in Table 1 therefore denote the counting unit within each dataset's single assigned role, not a within‐subject split. This source‐level, cross‐dataset (and, for UH‐10, cross‐device) protocol provides a more stringent generalization test than random within‐dataset splitting and follows recommended practice for avoiding data leakage in medical‐imaging machine learning^45^, ensuring that each paired comparison is computed on held‐out inputs not used to fit any model and that the inferential analysis contrasts methods on independent rather than training‐correlated cases. Although the datasets span different anatomical regions and acquisition settings, speckle reduction is a low‐level restoration task governed by scatterer statistics and the point‐spread function rather than organ‐specific semantics,^2^, ^8^, ^9^ so the simulator‐based training uses this anatomical diversity deliberately to encourage anatomy‐agnostic generalization. Moreover, the simulator randomizes the point‐spread‐function widths and the speckle‐variance parameter to span the speckle statistics of different transducer frequencies and configurations; this domain‐randomization strategy^46^ places the evaluation data within the trained distribution rather than treating it as out‐of‐distribution extrapolation. Cross‐anatomy and cross‐device performance is then verified empirically on the external UH‐10 bladder set rather than assumed. The exact dataset roles are summarized in Table 1

**TABLE 1:** Summary of datasets and their roles in the study.

Dataset	Data type	Partition unit	Training	Validation	External evaluation
BUSI^43^	B‐mode image	per‐image	780	0	0
HC18^44^	B‐mode image	per‐image	999	0	0
PICMUS^38^	raw acquisition	per‐acquisition	0	4	0
CUBDL^39^	raw acquisition	per‐acquisition	0	73	0
UH‐10	B‐mode image	per‐image	0	0	94

**FIGURE 7:**
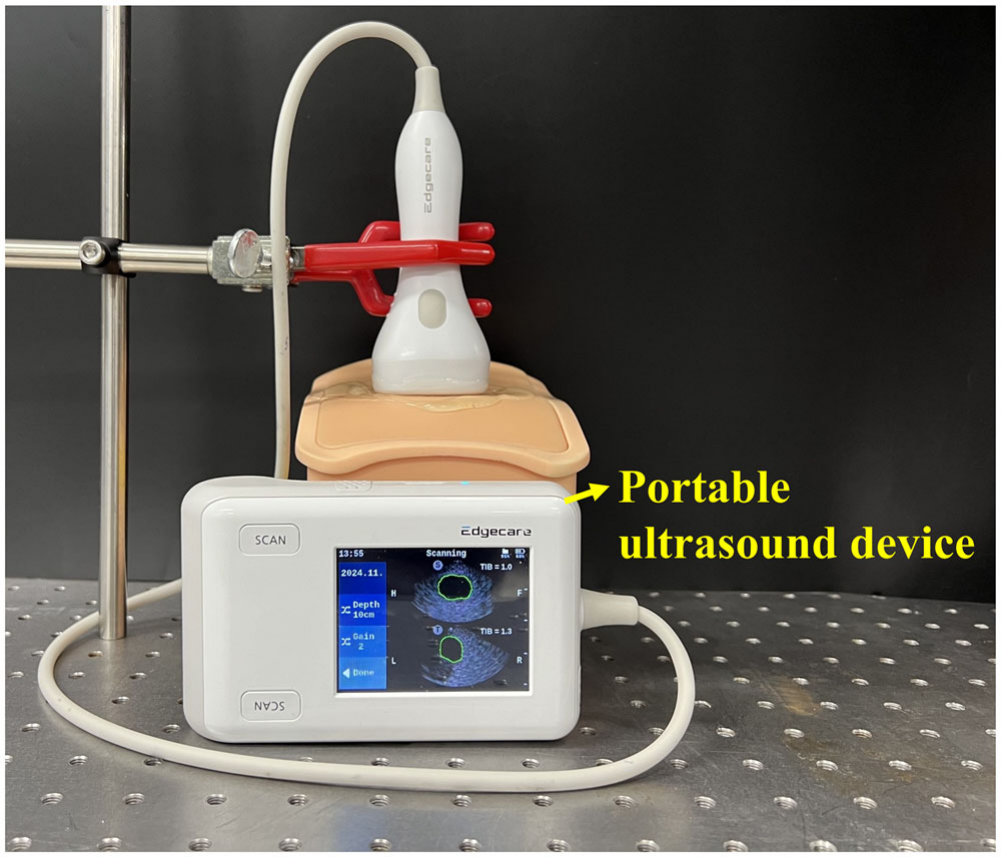
Experimental setup demonstrating the portable ultrasound device used in this study. The proposed deep learning model was deployed on an evaluation board using the same SoC architecture as the portable ultrasound device.

To validate the proposed EdgeSRIE framework with US images acquired by a real portable US device, additional data obtained using the portable bladder scanner EdgeFlow UH‐10 (Edgecare Inc., Seoul, Republic of Korea) were utilized. The in vivo bladder data used in this study were obtained with approval from the Yonsei University Institutional Review Board (IRB No. 1‐2022‐0076). Figure 7 shows the portable scanner used in this study. The acquired images were used for offline comparative image‐quality evaluation across methods, whereas an evaluation board equipped with the same SoC as the scanner was used for runtime profiling.

Both conventional rule‐based filters and DL models were used as baselines, selected to span the principal despeckling paradigms and to bracket the quality‐versus‐cost trade‐off, thereby contrasting EdgeSRIE against both computationally heavy and lightweight alternatives. Each baseline is an established US despeckling method with a publicly described implementation, supporting a fair and reproducible comparison. The rule‐based filters represent the two dominant classical families: oriented speckle reducing anisotropic diffusion (OSRAD),^16^ an edge‐aware anisotropic‐diffusion filter, and optimized Bayesian nonlocal means (OBNLM),^20^ a patch‐similarity nonlocal‐means filter. The DL baselines represent complementary convolutional design choices: A dilated‐convolution autoencoder (DIAE)^26^, a U‐Net–based denoising network (DUNet)^26^, a batch‐renormalization U‐Net (BRUNet)^26^, and US‐Net (USNet)^27^, a lightweight network for joint speckle suppression and texture enhancement. DIAE,^26^ DUNet,^26^ BRUNet,^26^ and USNet^27^ were trained with a supervised scheme using the same simulator‐generated data source, whereas EdgeSRIE used the proposed unsupervised and self‐supervised training strategy. For the floating‐point training stages, AdamW^47^ was used with a learning rate of $1\times 10^{-4}$, a decoupled weight‐decay coefficient of $1\times 10^{-2}$, β1=0.9, β2=0.999, and $\varepsilon =1\times 10^{-8}$. For all methods, the final checkpoint was selected post hoc as the one maximizing ROI‐averaged validation‐CNR. EdgeSRIE is reported both in floating‐point APU mode and in its final 8‐bit DPU deployment mode, whereas the baselines are reported in floating‐point APU mode because they could not all be compiled into an equivalent single‐subgraph DPU configuration under the toolchain used in this study.

To assess each speckle reduction method quantitatively, contrast‐to‐noise ratio (CNR)^29^, ^48^ and speckle signal‐to‐noise ratio (SSNR)^48^ were calculated as speckle‐suppression metrics, because they quantify contrast enhancement and homogeneous‐region speckle reduction. Average gradient magnitude (AGM)^27^ was used as an edge‐gradient metric to evaluate boundary‐related detail preservation, and structural similarity index measure (SSIM)^27^ was used to quantify structural similarity between processed and original images. These metrics were selected to represent the main trade‐off in despeckling: stronger speckle suppression versus preservation of structural detail. The metrics are defined as follows:

(5) $$\mathrm{CNR}\;\left(R_{B},R_{C}\right)=20\cdot \log_{10}\left(\frac{\left|\mu_{R_{B}}-\mu_{R_{C}}\right|}{\sqrt{\sigma_{R_{B}}^{2}+\sigma_{R_{C}}^{2}}}\right)$$

(6) $$\mathrm{SSNR}\;\left(R\right)=\frac{\mu_{B}}{\sigma_{B}}\;$$

(7) $$\mathrm{AGM}\;=\frac{1}{N-1}\;\sum_{n=1}^{N-1}\left|I\left(n+1\right)-I\left(n\right)\right|$$

(8) $$\mathrm{SSIM}\;\left(f,g\right)=\frac{\left(2\mu_{f}\mu_{g}+c_{1}\right)\left(2\sigma_{fg}+c_{2}\right)}{\left(\mu_{f}^{2}+\mu_{g}^{2}+c_{1}\right)\left(\sigma_{f}^{2}+\sigma_{g}^{2}+c_{2}\right)}\;$$

where $R_{B}$ and $R_{C}$ are the background and cystic regions; μ, σ, and $\sigma_{fg}$ are the mean, standard deviation, and covariance of each region; f, and g are the predicted and original images; I and N are the intensity profile and profile length; and $c_{1}$ and $c_{2}$ are the standard SSIM stabilization constants, set to 6.5025 and 58.5225 to avoid numerical instability when the local mean or variance is small.

### 2.6 Statistical analysis

Inferential analysis was performed on the case‐matched evaluation set reported in the manuscript: Three display settings each for the PICMUS^38^ in vivo carotid‐long and CUBDL^39^ phantom cases, plus two representative UH‐10 cases (*n* = 8 paired observations). For each metric, EdgeSRIE was compared pairwise with OSRAD,^16^ OBNLM,^20^ DIAE,^26^ DUNet,^26^ BRUNet,^26^ and USNet^27^ using two‐sided Wilcoxon signed‐rank tests^49^ with *α* = 0.05. Holm correction^50^ was applied across the six baseline comparisons within each metric.

Practical significance was quantified using complementary effect‐size measures. We report two complementary effect sizes: the matched‐pairs rank‐biserial correlation^51^, which captures the direction‐consistency of the paired differences but reflects ranking direction rather than magnitude, and—because a rank‐based coefficient can appear large even when the absolute difference is small—the matched‐pairs Hedges' *g* (the small‐sample‐bias‐corrected standardized mean of the paired differences; correction factor $J=1-\frac{3}{\left(4\cdot df-1\right)}$, interpreted as small (|g|≥0.2), medium (|g|≥0.5), or large (|g|≥0.8),^52^ with the equivalent Cohen's *d_z_*. Statistical significance and the magnitude‐based Hedges’ *g* are interpreted jointly throughout. Relative improvement was computed as 100×(methodvalue−rawinputvalue)/rawinputvalue and is reported only for the unbounded suppression and gradient metrics (CNR, SSNR, and AGM), for which a linear percentage is meaningful. Because the structural similarity index measure (SSIM) is bounded to [0, 1] and compressed near its ceiling, a linear percentage distorts high‐fidelity reconstructions; SSIM is therefore summarized on its native scale using the absolute values in Table 2. The relative‐improvement table thus describes the unbounded metrics, whereas the Wilcoxon/Holm analysis provides the formal paired comparison.

**TABLE 2:** Main quantitative comparison on the four representative cases shown in Figures 8, 9, 10 (CUBDL phantom, PICMUS in vivo carotid‐long, UH10‐002, and UH10‐003). Higher values indicate better performance (↑). Bold values indicate the best value for each metric within each case; asterisks indicate Holm‐corrected significant difference versus EdgeSRIE; parentheses denote the matched‐pairs Hedges’ *g* effect‐size category (S, M, or L) from the *n* = 8 paired analysis.

Case	Method	Ref.	CNR↑	SSNR↑	AGM↑	SSIM↑
CUBDL^39^ phantom (Figure 8)	Input	—	11.69	6.55	13.90	1.00
	OSRAD	16	17.67*(L)	10.55*(L)	5.27*(L)	0.96*(L)
	OBNLM	20	18.03*(L)	17.20(S)	5.59(L)	0.96*(L)
	DIAE	26	20.99*(L)	14.74*(L)	4.78*(L)	0.93*(L)
	DUNet	26	20.05*(L)	14.09*(L)	5.46*(L)	0.91*(L)
	BRUNet	26	18.24*(L)	12.11*(L)	4.92*(L)	0.90*(L)
	USNet	27	17.46*(L)	10.44*(L)	6.06(M)	0.92*(L)
	EdgeSRIE	This work	**21.75**	**17.85**	**6.12**	0.94
PICMUS^38^ in vivo carotid‐long (Figure 9)	Input	—	8.97	4.35	11.39	1.00
	OSRAD	16	13.16*(L)	8.26*(L)	5.26*(L)	0.95*(L)
	OBNLM	20	12.09*(L)	**10.42(S)**	6.14(L)	0.96*(L)
	DIAE	26	12.27*(L)	7.76*(L)	5.74*(L)	0.92*(L)
	DUNet	26	14.96*(L)	8.81*(L)	5.08*(L)	0.91*(L)
	BRUNet	26	14.50*(L)	8.13*(L)	5.46*(L)	0.89*(L)
	USNet	27	11.40*(L)	5.25*(L)	7.26(M)	0.87*(L)
	EdgeSRIE	This work	**15.25**	8.91	**8.30**	0.93
UH10‐002 (Figure 10–2)	Input	—	13.78	7.80	7.31	1.00
	OSRAD	16	16.75*(L)	10.93*(L)	6.43*(L)	0.99*(L)
	OBNLM	20	17.96*(L)	12.37(S)	5.88(L)	0.99*(L)
	DIAE	26	17.53*(L)	10.92*(L)	7.43*(L)	0.98*(L)
	DUNet	26	17.48*(L)	11.07*(L)	7.25*(L)	0.98*(L)
	BRUNet	26	18.04*(L)	10.44*(L)	7.98*(L)	0.97*(L)
	USNet	27	15.17*(L)	8.22*(L)	6.46(M)	0.88*(L)
	EdgeSRIE	This work	**18.74**	**12.71**	**8.95**	0.98
UH10‐003 (Figure 10–1)	Input	—	9.15	5.44	7.93	1.00
	OSRAD	16	12.93*(L)	9.42*(L)	5.14*(L)	0.99*(L)
	OBNLM	20	13.71*(L)	10.46(S)	5.27(L)	0.99*(L)
	DIAE	26	14.64*(L)	10.88*(L)	5.43*(L)	0.98*(L)
	DUNet	26	14.78*(L)	9.27*(L)	6.79*(L)	0.99*(L)
	BRUNet	26	13.80*(L)	7.72*(L)	6.78*(L)	0.98*(L)
	USNet	27	10.66*(L)	6.32*(L)	5.89(M)	0.90*(L)
	EdgeSRIE	This work	**15.30**	10.84	**7.48**	0.98

## 3 RESULTS

### 3.1 Qualitative evaluation

**FIGURE 8:**
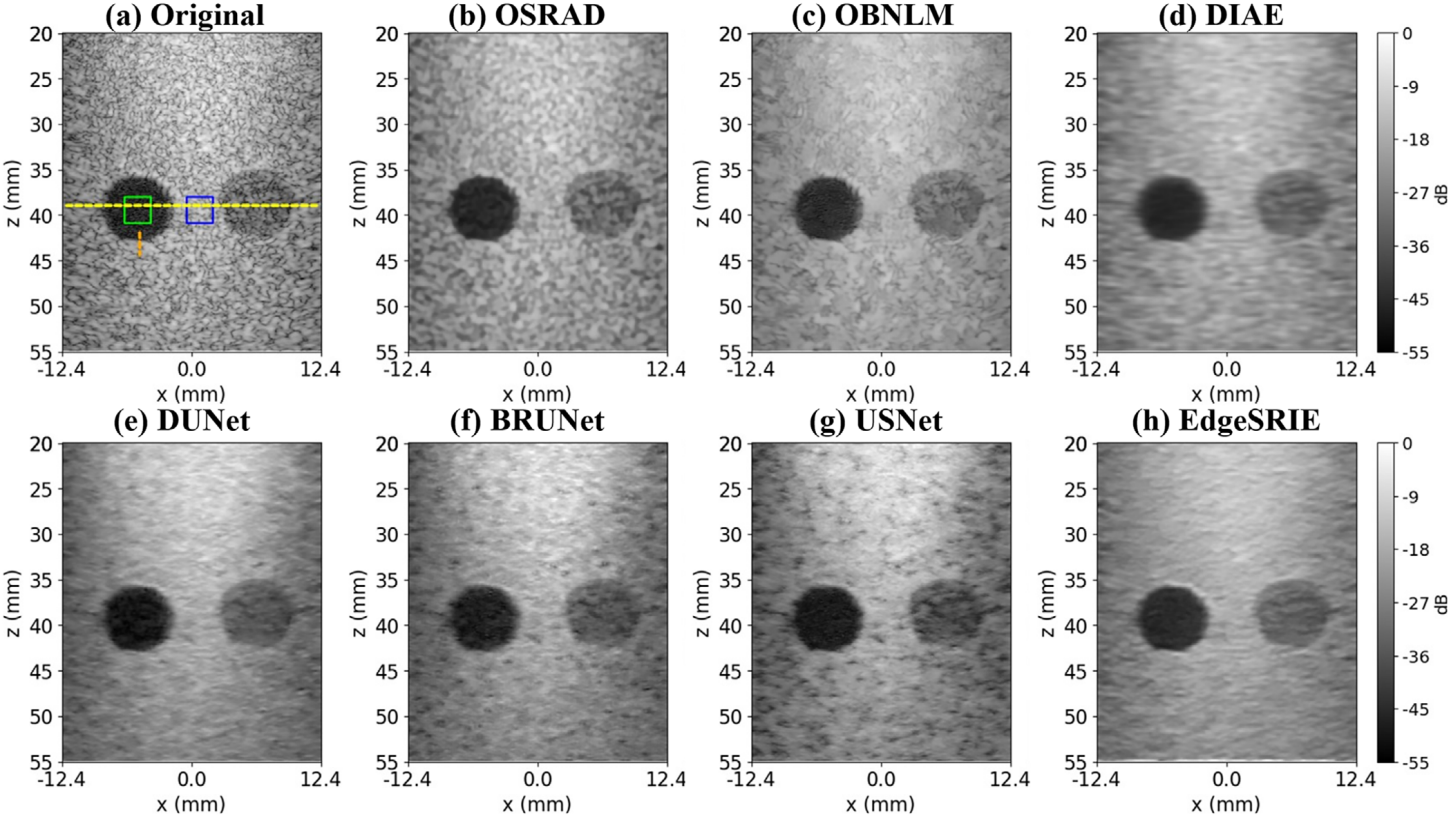
Comparative speckle‐reduction results on the representative CUBDL phantom case. (a) Original speckled B‐mode image with two circular inclusions. Speckle‐reduction results obtained using (b) OSRAD,^16^ (c) OBNLM,^20^ (d) DIAE,^26^ (e) DUNet,^26^ (f) BRUNet,^26^ (g) USNet,^27^ and (h) the proposed EdgeSRIE method. All images are displayed with the same lateral and axial axes and on the same logarithmic grayscale range from 0 to −55 dB. The yellow dashed line indicates the ROI used for lateral profile analysis, green and blue boxes denote ROIs for contrast measurements, and orange dashed lines indicate ROIs for AGM evaluation.

Figure 8 presents speckle reduction results obtained for an experimental phantom from the CUBDL dataset featuring two circular inclusions. Figure 8(a) shows the original speckled B‐mode image displayed on a logarithmic scale from 0 to −55 dB. The green and blue boxes indicate ROIs for contrast measurements, whereas the yellow dashed line marks the ROI for lateral profile analysis. Figures 8(b)–(g) display the results of OSRAD,^16^ OBNLM,^20^ DIAE,^26^ DUNet,^26^ BRUNet,^26^ and USNet,^27^ respectively, and Figure 8(h) illustrates the proposed EdgeSRIE result. The proposed method reduces background fluctuation while preserving the main inclusion boundaries, whereas the compared methods show differing degrees of residual speckle, smoothing, or artificial texture.

**FIGURE 9:**
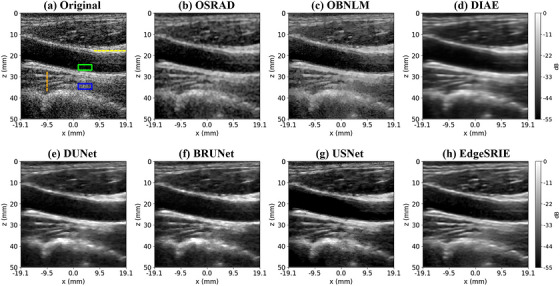
B‐mode images illustrating the longitudinal view of a carotid artery before and after speckle reduction using different methods. (a) Original ultrasound image, where the dashed yellow lines indicate lateral cross‐sectional profiles, the dashed orange line indicates an axial cross‐sectional profile, and the green and blue boxes mark the ROIs for contrast evaluation. Speckle‐reduction results using (b) OSRAD,^16^ (c) OBNLM,^20^ (d) DIAE,^26^ (e) DUNet,^26^ (f) BRUNet,^26^ (g) USNet,^27^ and (h) the proposed EdgeSRIE. All images use the same spatial axes and grayscale range from 0 to −55 dB.

Figure 9 presents comparative speckle reduction results for an in vivo carotid‐long image from the PICMUS dataset. Figure 9(a) shows the original B‐mode image on a logarithmic scale from 0 to −55 dB. The dashed yellow lines indicate lateral profiles, the dashed orange line marks the axial profile, and the green and blue boxes represent ROIs for quantitative contrast assessment. Figures 9(b)–(g) depict results obtained using OSRAD,^16^ OBNLM,^20^ DIAE,^26^ DUNet,^26^ BRUNet,^26^ and USNet,^27^ respectively, whereas Figure 9(h) displays the proposed EdgeSRIE result. EdgeSRIE reduces speckle while retaining major vascular boundaries, although the final interpretation remains an image‐quality assessment rather than a reader‐based clinical validation.

**FIGURE 10:**
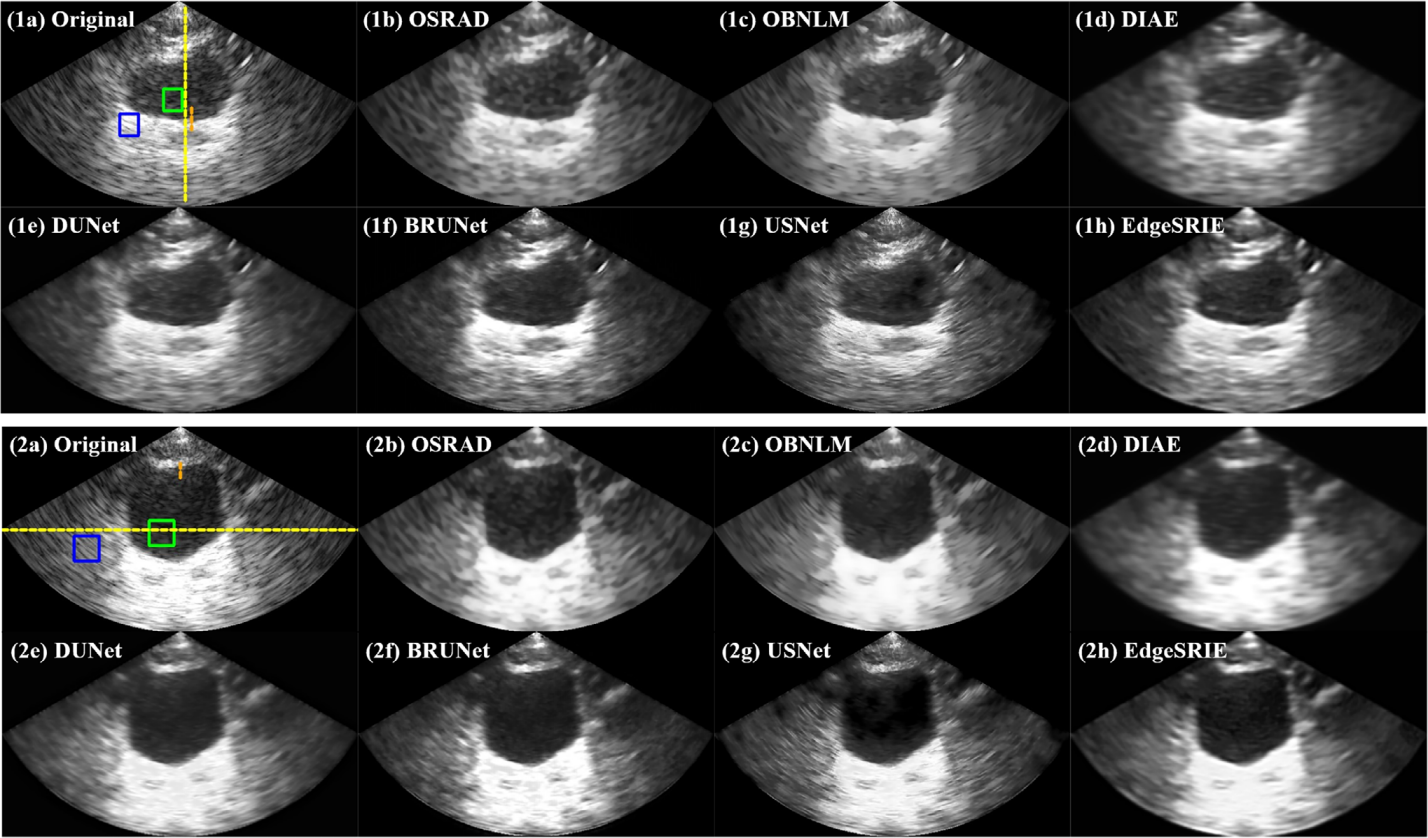
Speckle‐reduction results for two representative bladder ultrasound frames acquired with the portable EdgeFlow UH‐10 device. Rows correspond to two cases, and columns show the original image followed by OSRAD,^16^ OBNLM,^20^ DIAE,^26^ DUNet,^26^ BRUNet,^26^ USNet,^27^ and EdgeSRIE. Dashed yellow and orange lines indicate profiles used for intensity and AGM analysis, and green and blue boxes indicate ROIs used for contrast evaluation. The same display range is used within each case.

Figure 10 presents representative results from two bladder US frames acquired using the portable EdgeFlow UH‐10 device. The original images show hypoechoic bladder regions surrounded by speckled soft tissue. Across both cases, EdgeSRIE reduces speckle in homogeneous regions while retaining bladder boundary transitions. These examples provide external portable‐device image‐quality evaluation; they do not constitute a formal reader study or task‐based clinical validation.

### 3.2 Profile comparison

**FIGURE 11:**
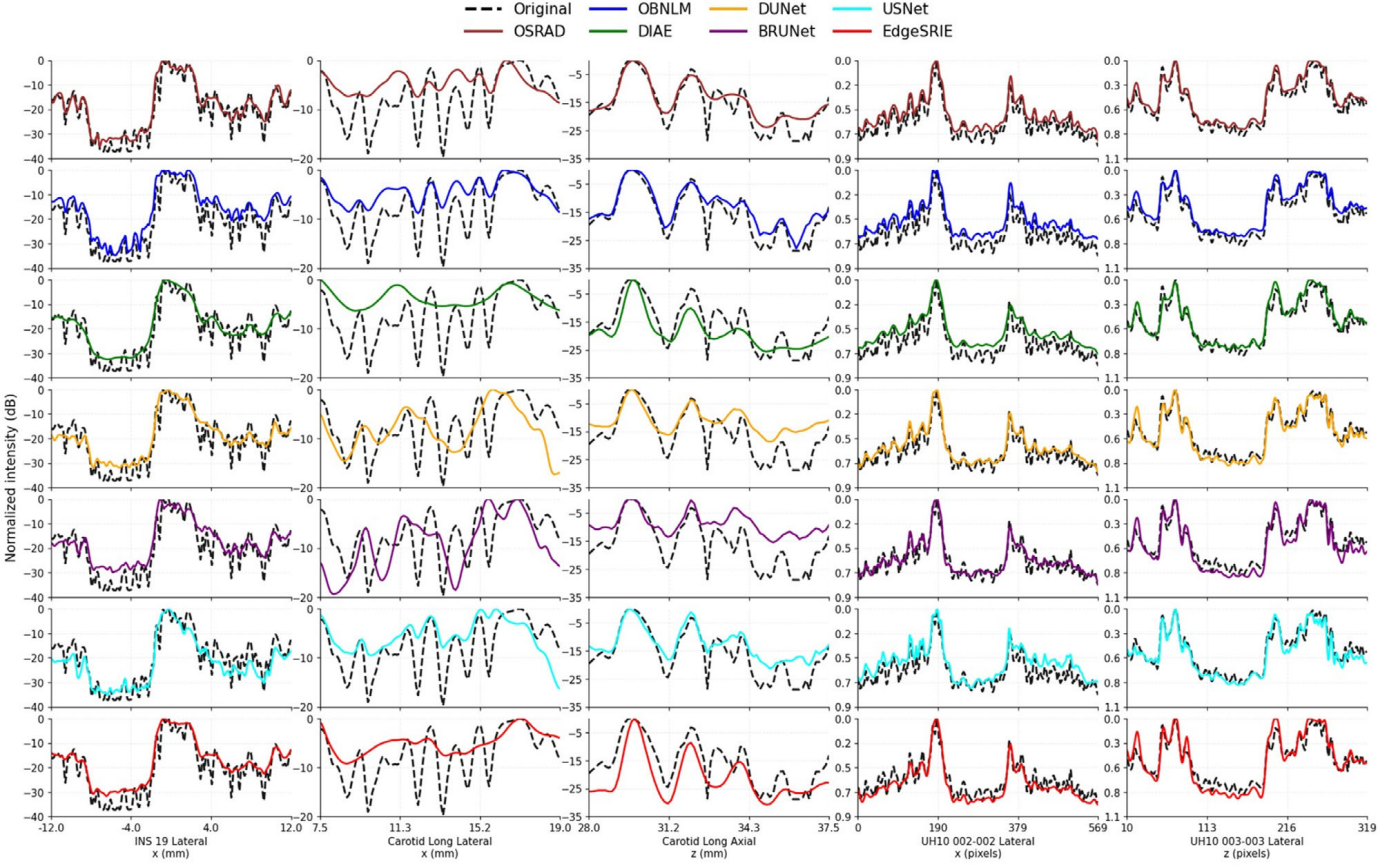
Method‐specific profile comparison for the representative CUBDL phantom, PICMUS in vivo carotid‐long, and portable‐device cases shown in Figures 8, 9, 10. Columns correspond to lateral or axial profiles sampled from each case, and rows correspond to OSRAD,^16^ OBNLM,^20^ DIAE,^26^ DUNet,^26^ BRUNet,^26^ USNet,^27^ and EdgeSRIE. In each panel, the original profile is shown as a black dashed line and the processed profile as a solid line using matched axes.

Figure 11 summarizes method‐specific intensity profiles from the CUBDL^39^ phantom, PICMUS^38^ carotid‐long, and portable‐device bladder cases. By showing one processed profile against the corresponding original profile in each panel, the revised presentation makes residual fluctuations, flattening near steep transitions, and preservation of boundary‐related slopes easier to compare across methods. EdgeSRIE shows a balanced profile pattern, reducing local fluctuations while retaining the main transition shape.

### 3.3 Quantitative evaluation

Table 2 summarizes the main quantitative comparison for the four representative cases shown in Figures 8, 9, 10: the CUBDL phantom, the PICMUS in vivo carotid‐long image, and two UH‐10 portable‐device bladder cases. Higher values indicate better performance for all metrics. Bold values identify the best value within each case and metric; asterisks indicate Holm‐corrected significant differences versus EdgeSRIE, and parentheses denote Hedges’ *g* effect‐size categories (S, M, or L).

Table 3 summarizes relative improvement over the raw input across the same representative cases. EdgeSRIE delivers the largest mean CNR improvement (64.9 ± 21.0%) and the smallest mean AGM decrease (−16.6 ± 33.2%). Its SSNR (109.8 ± 45.8%) improvement is in the OBNLM^20^‐led range, whereas SSIM remains slightly lower than OSRAD^16^ and OBNLM^20^ on its native [0, 1] scale. Thus, the descriptive results support a balanced trade‐off rather than uniform superiority on every metric.

**TABLE 3:** Relative improvement (%) over the raw input across the four representative cases in Table 2 (mean ± standard deviation). The upward arrow (↑) indicates that higher values are desirable for CNR and SSNR; AGM decreases are interpreted as smaller‐is‐better losses.

Method	Ref.	CNR↑(%)	SSNR↑(%)	AGM↑(%)
OSRAD	16	40.2 ± 13.1	66.1 ± 20.9	−40.8 ± 22.2
OBNLM	20	42.3 ± 11.5	**113.3 ± 46.7**	−39.7 ± 17.2
DIAE	26	50.9 ± 23.6	85.9 ± 36.0	−36.3 ± 28.9
DUNet	26	56.7 ± 20.3	82.5 ± 33.0	−32.8 ± 29.7
BRUNet	26	49.9 ± 13.4	61.9 ± 27.9	−30.5 ± 33.9
USNet	27	25.8 ± 17.2	25.4 ± 23.5	−32.5 ± 18.9
EdgeSRIE	This work	**64.9 ± 21.0**	109.8 ± 45.8	−**16.6 ± 33.2**

### 3.4 Statistical evaluation

In the *n* = 8 case‐matched analysis, EdgeSRIE was Holm‐corrected significantly higher than all six baselines in CNR, with large effect sizes throughout (Hedges’ *g* = 0.90–4.95). For SSNR, EdgeSRIE was significantly higher than OSRAD,^16^ DIAE,^26^ DUNet,^26^ BRUNet,^26^ and USNet^27^ (*g* = 0.92–2.81, all large), whereas the difference from OBNLM^20^ was not significant and small in magnitude (*g* = −0.29). For AGM, EdgeSRIE was significantly higher than OSRAD,^16^ DIAE,^26^ DUNet,^26^ and BRUNet^26^ (*g* = 1.31–2.92, all large); the comparisons with OBNLM^20^ and USNet^27^ did not reach Holm‐corrected significance but showed large and medium effect sizes favoring EdgeSRIE (*g* = 0.95 and 0.68, respectively). For SSIM, EdgeSRIE was significantly higher than the four DL baselines (*g* = 1.02–1.53, all large) but significantly lower than OSRAD^16^ and OBNLM^20^ (*g* = −2.91 and −2.97, large). The corresponding matched‐pairs Hedges’ *g* effect size for each EdgeSRIE‐versus‐baseline comparison is summarized in Table 4.

**TABLE 4:** Matched‐pairs Hedges' g for EdgeSRIE versus each baseline (*n* = 8). Positive values favor EdgeSRIE; asterisks denote Holm‐corrected significance (*p* < 0.05). Thresholds: Small |g|≥0.2, medium |g|≥0.5, large |g|≥0.8.

Method	CNR	SSNR	AGM	SSIM
OSRAD^16^	2.47*	0.92*	1.81*	−2.91*
OBNLM^20^	2.22*	−0.29	0.95	−2.97*
DIAE^26^	0.90*	1.32*	2.92*	1.02*
DUNet^26^	1.04*	1.05*	1.31*	1.05*
BRUNet^26^	1.14*	1.25*	1.51*	1.53*
USNet^27^	4.95*	2.81*	0.68	1.18*

### 3.5 Execution time analysis on a portable US device

**FIGURE 12:**
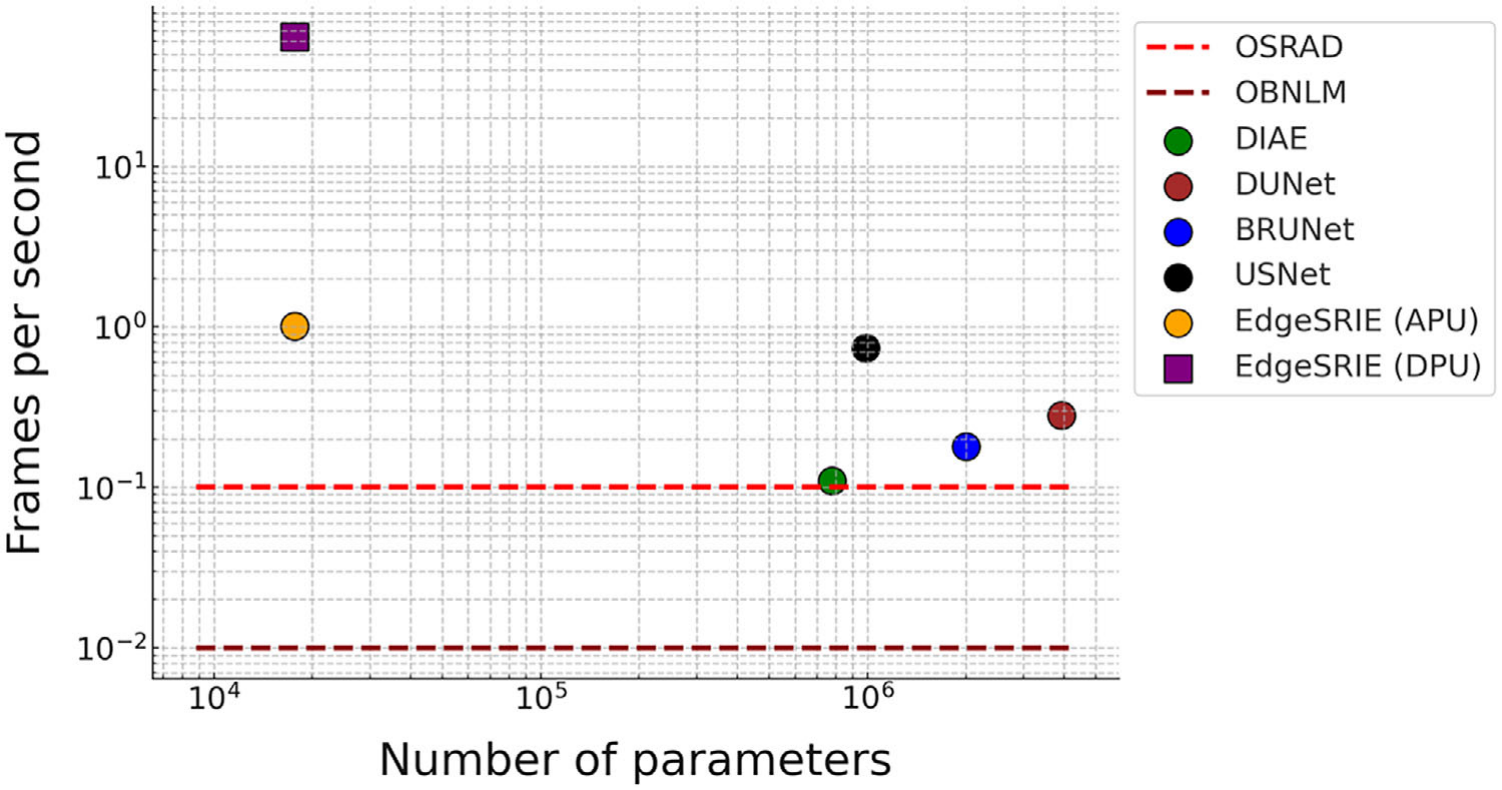
Comparison of model complexity and execution speed on the target low‐resource SoC platform. The x‐axis indicates the number of parameters, and the y‐axis indicates frames per second on a log‐log scale. OSRAD^16^ and OBNLM^20^ were profiled on the APU, the compared deep learning baselines were profiled in floating‐point mode, and EdgeSRIE is shown both in floating‐point mode and in its final 8‐bit deployed configuration.

Figure 12 and Table 5 summarize computational complexity and runtime on the target low‐resource SoC. In this study, real‐time operation refers to at least approximately 25–30 frames/s. The rule‐based methods and DL baselines were profiled in floating‐point execution on the APU, whereas EdgeSRIE is additionally reported in its 8‐bit post‐training‐quantized deployment on the DPU. All APU‐mode values fall below the real‐time threshold (0.01–1.01 frames/s), while EdgeSRIE reaches 64.10 frames/s in its deployed configuration. The reported frame rate corresponds to the on‐device inference latency of the deployed model, measured on an evaluation board carrying the same SoC as the portable scanner; it therefore characterizes the speckle‐reduction and enhancement inference stage rather than the throughput of a fully integrated, end‐to‐end live imaging pipeline encompassing beamforming, envelope detection, log compression, and display.

**TABLE 5:** Computational complexity analysis of different methods in low‐resource SoC settings. Higher FPS values indicate better real‐time capability (↑), whereas fewer parameters and FLOPs indicate more efficient deployment (↓).

Methods	Processor	FPS↑	Parameters↓	FLOPs↓
OSRAD^16^	APU	0.10	X	X
OBNLM^20^	APU	0.01	X	X
DIAE^26^	APU	0.11	780.16K	25.2 G
DUNet^26^	APU	0.28	3.92 M	48.0 G
BRUNet^26^	APU	0.18	2.00 M	113.3 G
USNet^27^	APU	0.74	987.34K	2.02 G
EdgeSRIE	APU	1.01	17.67K	564.1 M
	DPU	64.10		

The deployed DPU result should be interpreted as a deployment‐oriented benchmark for the proposed pipeline rather than a fully hardware‐matched 8‐bit comparison across all methods, because the baseline networks could not all be compiled into the same single‐subgraph DPU configuration under the tool chain used in this study.

### 3.6 Ablation study

To assess the contribution of the main components, we performed a three‐way ablation on the representative CUBDL phantom case: the integrated floating‐point model, the model without the deblurring branch, and the final 8‐bit post‐training‐quantized deployment model. This comparison isolates the effect of the deblurring branch and the deployment‐stage quantization effect.

The ablation indicates that the deblurring branch mainly supports local transition restoration and structural fidelity after despeckling, whereas the despeckling branch drives fluctuation suppression. The 8‐bit deployed model remains close to the floating‐point result while enabling the runtime gain reported in Table 5; therefore, post‐training quantization primarily contributes deployment efficiency rather than image‐quality improvement.

### 3.7 Training convergence

**FIGURE 13:**
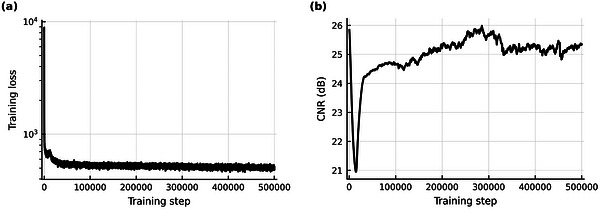
Training convergence of the unsupervised despeckling branch. (a) Training loss (log scale) and (b) ROI‐averaged held‐out CNR on a representative acquisition versus training step. The loss settles to a stable plateau and the held‐out CNR plateaus without sustained decline, indicating convergence with no sustained late‐stage decline in the held‐out metric.

Figure 13 shows the convergence behavior of the unsupervised despeckling branch, whose checkpoint governs the speckle‐suppression performance of the integrated model. The training loss decreased steeply by approximately 93% within the first 50 000 steps and then settled onto a low, stable plateau (Figure 13(a)), indicating sufficient capacity to fit the training objective and convergence rather than underfitting. To assess generalization, the contrast‐to‐noise ratio (CNR) was monitored on a held‐out acquisition excluded from training; after a brief transient, this validation‐CNR rose and then fluctuated around approximately 25 dB with a flat trend and no decline trend over the monitored training horizon (Figure 13(b)). The final despeckling checkpoint was selected as the one achieving the highest ROI‐averaged validation‐CNR, and the same criterion was applied to all baselines.

## 4 DISCUSSION

The Discussion is organized according to the three objectives stated in the Introduction and the principal limitations of the present study. This structure is intended to link the reported results directly to the expected accomplishments while avoiding overstatement of clinical efficacy.

### 4.1 Achievement of objective 1: Practical training without clean ground‐truth references

The first objective was to develop an US enhancement framework that does not require perfectly clean reference images for training. This objective is addressed by the simulator‐supported unsupervised despeckling branch and the self‐supervised deblurring branch. The former learns from multiple stochastic speckle realizations that share the same underlying structure, and the latter restores synthetically degraded despeckled references. These design choices reduce dependence on paired clean targets, although generalization must still be verified empirically across independent acquisitions and device conditions, as evaluated with PICMUS,^38^ CUBDL,^39^ and UH‐10 data.

### 4.2 Achievement of objective 2: Balance between speckle suppression and structural detail preservation

**FIGURE 14:**
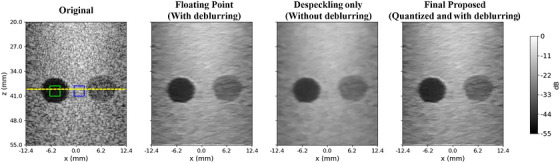
Component and deployment ablation on the representative CUBDL phantom case. From left to right: original image, integrated floating‐point model, model without the deblurring branch, and final post‐training‐quantized deployed model. All images use the same grayscale range.

**FIGURE 15:**
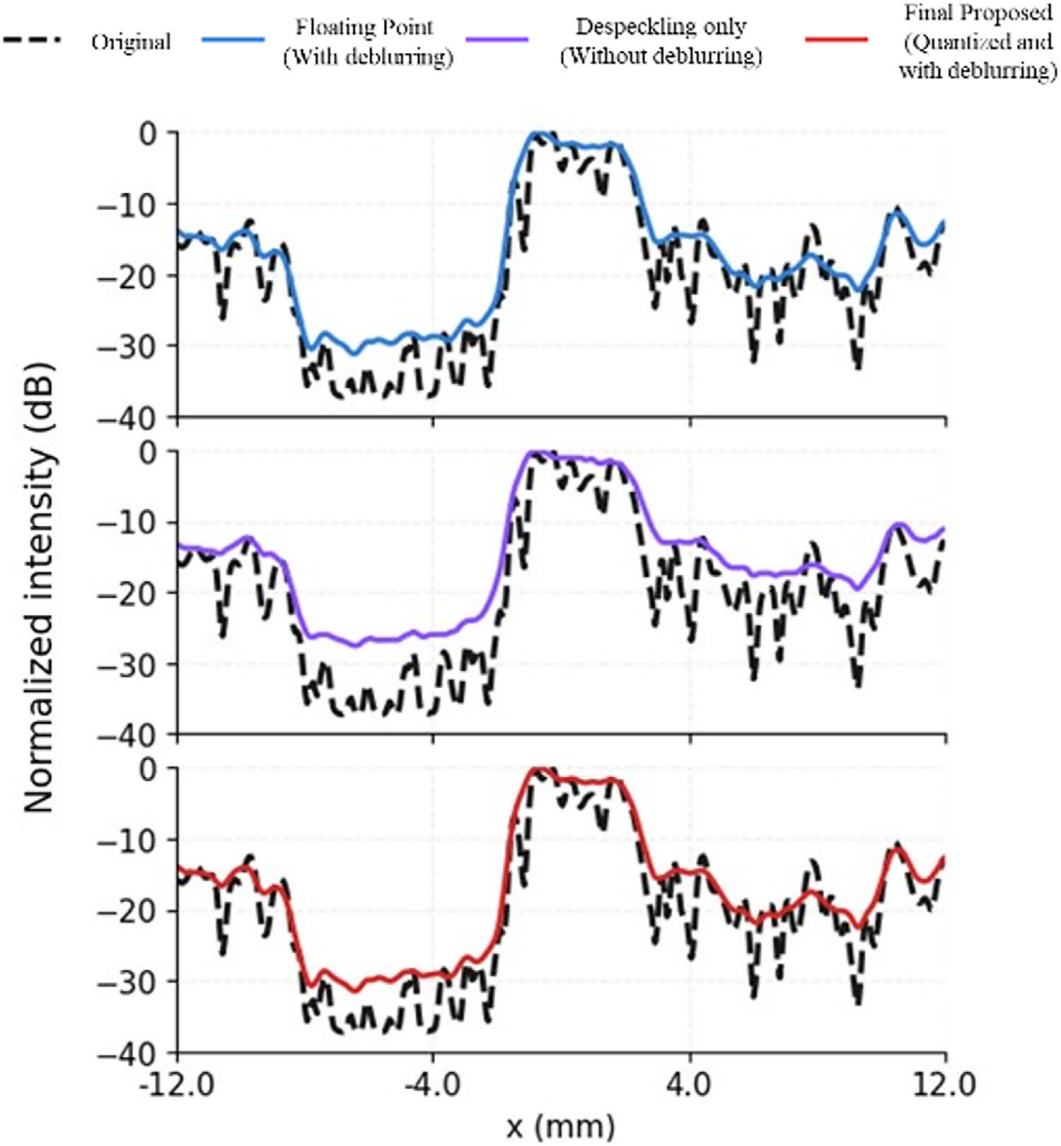
Profile comparison for the component and deployment ablation shown in Figure 14. The original signal is shown as a black dashed line and the model output as a solid line; matched axes highlight preservation of the main transition and the effect of the deblurring branch on local detail restoration.

The second objective was to balance speckle suppression with preservation of structural detail. Quantitatively, EdgeSRIE achieved the largest mean CNR improvement and the smallest mean AGM decrease over the raw input, with SSNR gains in the OBNLM^20^‐led range, whereas SSIM remained slightly below the rule‐based filters on its native [0, 1] scale (Tables 2 and 3; Figures 8, 9, 10, 11). Each paired comparison is interpreted below using both Holm‐corrected significance (Section III.D) and the Hedges’ *g* effect size (Table 4), with strengths and deficiencies linked to the component ablation (Section III.F; Figures 14, 15).

Relative to the DL baselines (DIAE,^26^ DUNet,^26^ BRUNet,^26^ and USNet^27^), EdgeSRIE was Holm‐significantly higher in CNR, SSNR, and SSIM against all four baselines, and in AGM against DIAE,^26^ DUNet,^26^ and BRUNet^26^ (the AGM comparison with USNet^27^ is considered among the non‐significant findings below); the corresponding effect sizes confirm these advantages are of practical, not merely directional, importance (Table 4). The ablation analysis explains this profile mechanistically: The unsupervised despeckling branch drives homogeneous‐region fluctuation suppression, which underlies the CNR and SSNR gains, whereas the self‐supervised deblurring branch restores local transitions after despeckling, which preserves boundary gradients and accounts for the comparatively small AGM loss (Section III.F; Figures 14, 15). Because the supervised DL baselines optimize a single denoising objective without an explicit transition‐restoration stage, they tend to over‐smooth boundary detail, consistent with their larger AGM decreases in Table 3.

Relative to the rule‐based filters (OSRAD^16^ and OBNLM^20^), the comparison is intentionally mixed and constitutes the central trade‐off of the framework. EdgeSRIE was Holm‐significantly higher in CNR than both filters but Holm‐significantly lower in SSIM (Table 4). This unfavorable SSIM comparison is interpreted physically rather than as a defect: because SSIM is computed against the speckled original, filters such as OBNLM^20^ that smooth strongly toward low‐frequency structure retain higher similarity to that input, whereas EdgeSRIE deliberately alters fine speckle texture to enhance contrast and preserve boundary gradients. The same over‐smoothing that raises rule‐based SSIM is reflected in their larger AGM decreases (Table 3), so the higher rule‐based SSIM and the higher EdgeSRIE AGM describe two sides of the same suppression‐versus‐detail trade‐off rather than a uniform quality ranking.

Two comparisons that did not reach Holm‐corrected significance are interpreted explicitly. First, EdgeSRIE and OBNLM^20^ did not differ significantly in SSNR (Hedges’ *g* = −0.29); this is expected on physical grounds, because both methods are strong homogeneous‐region speckle suppressors and therefore converge on similar SSNR, so the result represents a genuine equivalence rather than an underpowered null. Second, for AGM, the comparisons with OBNLM^20^ and USNet^27^ did not reach Holm‐corrected significance despite effect sizes favoring EdgeSRIE (Hedges’ *g* = 0.95 and 0.68, respectively); here the non‐significance reflects the limited paired sample (*n* = 8) combined with the conservatism of the Holm correction, so the effect size, rather than the *p*‐value alone, conveys the practically relevant gradient‐preservation advantage. Interpreting these cases jointly through significance and effect size avoids both over‐interpreting direction‐consistent but small differences and dismissing practically meaningful differences that do not survive a conservative significance threshold. Overall, therefore, EdgeSRIE should be understood as a balanced operating point for suppression‐oriented enhancement with boundary‐gradient preservation rather than a method that is uniformly superior on every metric.

### 4.3 Achievement of objective 3: Real‐time deployment on a low‐resource portable platform

The third objective was to demonstrate real‐time deployment on low‐resource portable hardware. With 17.67K parameters and successful 8‐bit post‐training quantization, EdgeSRIE reached 64.10 frames/s on the target DPU configuration (Table 5; Figure 12). This result reflects the combination of compact architecture, quantization, and single‐subgraph deployment, and should be interpreted as a deployment‐oriented benchmark rather than a fully hardware‐matched 8‐bit comparison across all baselines.

### 4.4 Limitations and Considerations

Several limitations remain. First, the present study was not designed to establish diagnostic efficacy; no expert reader study, diagnostic interpretation task, or expert‐generated annotation was used as an outcome measure. Second, the main comparative results are based on representative case‐matched evaluations, and broader generalization will require larger multicenter datasets spanning additional anatomies, scanner settings, rare pathologies, and operator‐dependent acquisition patterns. Third, the unfavorable SSIM comparison against OSRAD^16^ and OBNLM^20^ indicates that future work should explore explicit structural‐fidelity or perceptual constraints, task‐based evaluation, and temporal consistency for dynamic US. Fourth, the reported real‐time performance reflects on‐device inference throughput measured on an evaluation board rather than validated end‐to‐end operation within a live, fully integrated imaging pipeline on the production scanner; integrating EdgeSRIE into the complete acquisition‐to‐display pipeline and confirming sustained real‐time operation under live streaming remain important future work.

Future work may also extend EdgeSRIE to advanced beamforming methods, temporally consistent video US processing, and volumetric US applications such as 3D obstetric imaging or real‐time surgical guidance. In addition, motion artifacts arising from probe movement, physiological motion, and tissue displacement during handheld point‐of‐care scanning constitute another source of image degradation; incorporating explicit motion estimation or motion‐compensated processing is therefore a worthwhile direction for subsequent extensions of the framework.

## 5 CONCLUSION

In conclusion, Edge Speckle Reduction and Image Enhancement (EdgeSRIE) combines simulator‐supported unsupervised despeckling, self‐supervised deblurring, and an 8‐bit deployment pipeline for portable US systems. Across CUBDL^39^ phantom, PICMUS in vivo,^38^ and portable‐device evaluations, the framework improved contrast and speckle homogeneity while preserving boundary‐related gradients. Its compact architecture enabled real‐time execution on the target low‐resource hardware. Within the scope of this study, these findings support EdgeSRIE as a feasible deployment‐oriented approach for portable US image enhancement; larger and more diverse clinical evaluations are needed to establish broader generalizability and clinical utility.

## CONFLICT OF INTEREST STATEMENT

The authors declare that they have no conflict of interest.
